# Identification of Rad51 as a prognostic biomarker correlated with immune infiltration in hepatocellular carcinoma

**DOI:** 10.1080/21655979.2021.1938470

**Published:** 2021-06-11

**Authors:** Hao Xu, Chen Xiong, Yuan Chen, Chi Zhang, Dousheng Bai

**Affiliations:** a Yangzhou University Medical College, Yangzhou, Jiangsu, P.R. China; b Dalian Medical University, Dalian, Liaoning, P.R. China; c Department of Hepatobiliary Surgery, Clinical Medical College, Yangzhou University, Yangzhou, Jiangsu, P.R. China

**Keywords:** Hepatocellular carcinoma, immune infiltration, ICGC, TCGA, DNA repair gene

## Abstract

Rad51, a DNA-repair-related gene, has been reported to be involved in multiple cancers. However, its link with immune infiltration in liver cancer still unknown. Therefore, more research into the roles and activities of Rad51 in hepatocellular carcinoma (HCC) is required. The International Cancer Genome Consortium (ICGC) was used to identify the DNA repair gene Rad51, and has been proved to be overexpressed in HCC patients. We plotted the Kapan-Meier curve, demonstrating that patients with high expression of Rad51 have a poor prognosis. By analyzing the patient data, we discovered that high expression of Rad51 in HCC is linked to clinical stage, pathological T stage, grade, and age. Rad51 was found to be an independent prognostic factor for HCC patients using the multivariate cox model. Moreover, Rad51 expression was found to be associated with the infiltration of immune cells (B cells, CD4 + T cells, CD8 + T cells, neutrophils, macrophages, and dendritic cells) and was intimately linked to the expression of immune cell markers in HCC. Through the analysis of differentially coexpressed genes (DCGs) of Rad51, GO and KEGG enrichment analyses suggested that the expression level of Rad51 might be relevant to neuroactive ligand-receptor interactions, the cell cycle, DNA replication, homologous recombination, oocyte meiosis, and the Fanconi anemia pathway. These findings indicated that Rad51 is a valuable biomarker for the prognosis of patients with liver cancer and that its expression has a significant correlation with immune infiltrations.

**Abbreviations:** HCC: hepatocellular carcinoma; ICGC: International Cancer Genome Consortium TCGA: The Cancer Genome Atlas; TIMER: Tumor Immune Estimation Resource; CAF: Cancer-associated fibroblast; GEPIA: Gene Expression Profiling Interactive Analysis; GSEA: Gene set enrichment analysis; OS: overall survival; PFS: progression-free survival; RFS: relapse-free survival; DSS: disease-specific survival. Partial cor: partial correlation coefficient; HPA: Human Protein Atlas; GO: Gene Ontology; KEGG: Kyoto Encyclopedia of Genes and Genomes; CAF: Cancer-associated fibroblast; DCGs: differentially co-expressed genes

## Graphical Abstract

**Figure:**
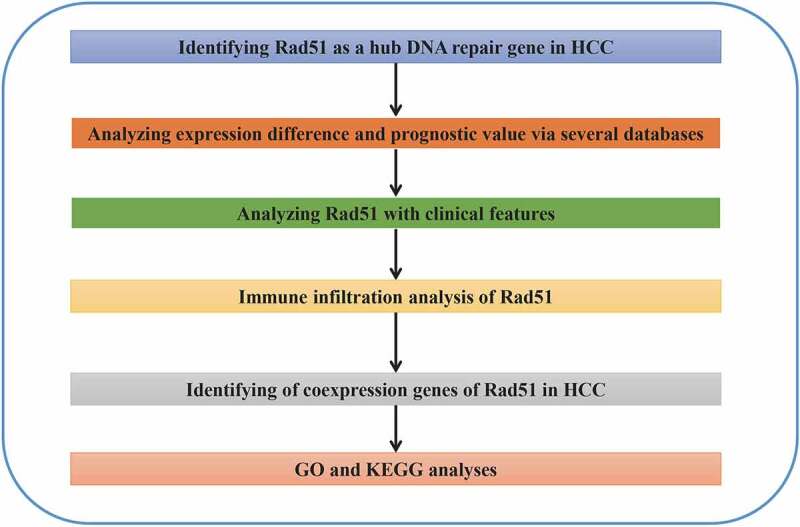

## Introduction

HCC is a malignant tumor of the digestive tract [1]. Surgical resection, liver transplantation, transhepatic arterial chemotherapy (TACE), and systemic therapy are all the therapeutic choices for patients with liver cancer [2]. Early-stage patients may benefit from surgical resection, but chemotherapy is preferred for patients with unresectable and advanced disease [3]. In fact, chemotherapy does not benefit all the patients with HCC, and the survival rate is still unsatisfactory [4]. Recently, according to the reports, immunotherapy has shown to be particularly promising for patients with liver cancer [5–7]. Consequently, it is crucial to seek novel possible biomarkers and potential targets for immunotherapy.

Many factors, according to the study, has been identified as contributing to poor efficacy of chemotherapy, including dysregulation of mitophagy [8], overexpression of drug efflux pumps [9], and hyperactivity of the DNA repair system [10]. The DNA damage repair system is an important mechanism for maintaining the stability of genetic material. Excessive DNA repair system activity leads to enhanced DNA repair ability and poor chemotherapy effects [11]. For instance, Ku70, a DNA repair gene, suppresses cell proliferation via interacting with the FOXO4 pathway [12].

In this study, by digging the ICGC and TCGA databases, we investigated the association among the Rad51 expression and clinical characteristics in patients with liver cancer. Afterward, we demonstrated the clinical prognostic value of Rad51 in HCC and identified that Rad51 could be a critical DNA repair gene associated with immune infiltration.

## Methods

### Data and information

Overall the information of 243 HCC tissues and 202 normal tissues were acquired from the International Cancer Genome Consortium (ICGC)(https://daco.icgc.org/) [13]. The identification of differentially expressed genes with done with |logFC| > 2. Then a total of 150 genes involved in DNA repair were retrieved by the ‘HALLMARK_DNA_REPAIR’ gene set in the Gene set enrichment analysis (GSEA) (http://www.gsea-msigdb.org/gsea/msigdb/cards/HALLMARK_DNA_REPAIR.html). We acquired ZWINT and Rad51 for further study after survival analysis of the screened differentially expressed DNA repair genes.

### Rad51 expression level and prognostic value in public datasets

The expression profiles and clinicopathological information, involving 374 HCC samples and 50 normal samples, were acquired from TCGA in order to validate the expression of RAD51 in HCC. We examined the expression of Rad51 in the ‘Expression DIY’ module of the GEPIA database (http://gepia.cancer-pku.cn/index.html) [14]. OS, PFS, RFS, and DSS were calculated through Kaplan-Meier plotter (http://kmplot.com/analysis/) [15]. Moreover, the protein levels of Rad51 in HCC were assessed by The Human Protein Atlas (https://www.proteinatlas.org/) [16]. Then the association between Rad51 expression and clinical features was assessed using the ‘ggpubr’ package and Perl language. Finally, we performed the univariate and multivariate Cox regression analyses in order to further investigate the prognostic value of Rad51 in HCC.

### TIMER database analysis

TIMER, a comprehensive platform, is employed to analyze immune infiltration across multiple cancer types (https://cistrome.shinyapps.io/timer/) [17]. We evaluated Rad51 expression in HCC and its correlation with the numbers of immune cells. Then, in order to reveal the impact of Rad51 on the markers of immune cells, a correlation analysis was conducted.

### CIBERSORT database analysis

Based on the expression level of Rad51, the 374 samples obtained from the TCGA-LIHC cohort were categorized into two groups: high expression and low expression. The difference in immune cell infiltration between two groups was further investigated using the CIBERSORT database, and the results were boxplot using the ‘ggpubr’ packages in R software.

### Identification of DCGs of Rad51 in HCC

A total of 19,921 coexpression genes were obtained from LinkedOmics database (https://www.biostars.org/p/287,820/) [18], and 11,781 genes were identified on the basis of the following thresholds: |correlation coefficient| > 0, FDR < 0.05. Then the ‘limma’ R package was employed to filter the DCGs with |logFC| > 2, and the first 50 genes positively and negatively related to Rad51 were separately plotted by the ‘heatmap’ R package.

### GO and KEGG enrichment analyses

We used the DCGs to perform GO and KEGG via ‘clusterprofiler’ R software. Corrected P-values <0.05 were found to be statistically relevant.

### Statistical analysis

R software and Perl languages (https://www.perl.org/) were used to conduct all the statistical analyses. The expression data were normalized via log2 transformation. The survival analyses were completed by log-rank testing. The correlation of Rad51 to several biomarkers of immune infiltrations was calculated using Spearman’s correlations. The correlations of infiltrating immunecells were determined via the following guide for the value of partial cor: 0.00–0.19: ‘very weak’, 0.20–0.39: ‘weak’, 0.40–0.59: ‘moderate’, 0.60–0.79: ‘strong’, and 0.80–1.0: ‘very strong’[19].

## Results

Rad51 is an oncogene that has been involved in a variety of cancers. Rad51 expression was revealed to be closely related to immune infiltration and the expression of immune cell markers in HCC after a comprehensive analysis of multiple databases. Resultantly, it might be a novel promising biomarker for immunization therapy in HCC.

### Filtration of differentially expressed genes in HCC

**Figure 1.**
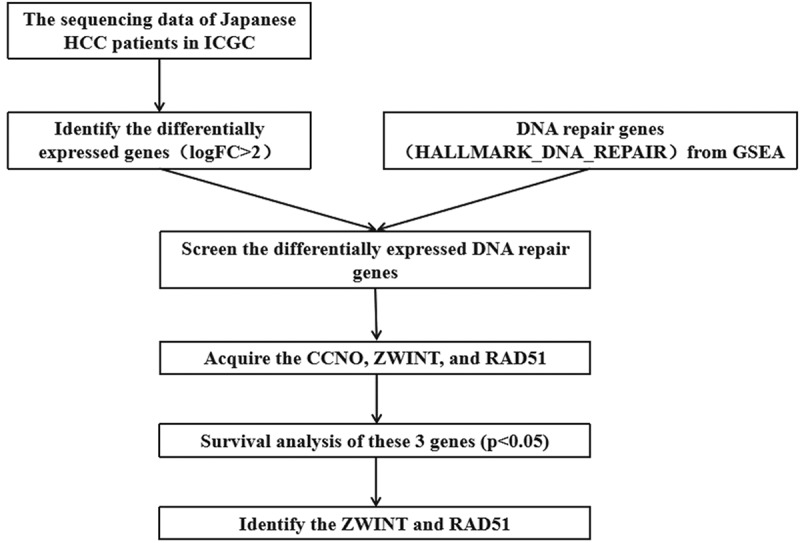
Flowchart of filtration process to identify target genes

**Figure 2.**
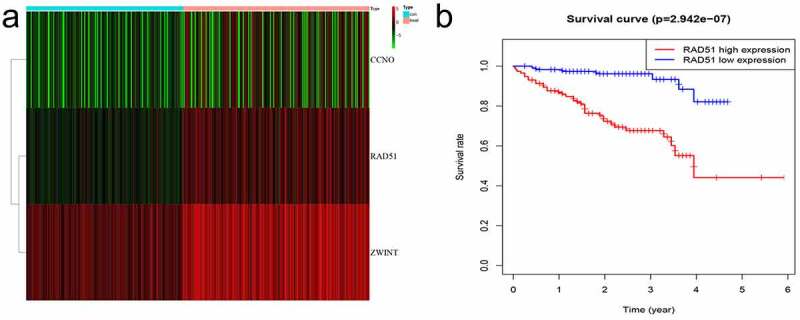
*Differentially expressed DNA repair genes*. A. Heatmap of the screened genes. B. Survival analysis of Rad51 in the patient with HCC from ICGC cohort

The differentially expressed genes acquired from ICGC were identified and then overlapped with genes related to DNA repair in order to obtain CCNO, Rad51, and ZWINT. The heatmap was employed to display the three differentially expressed genes (Figure 2 **A**). We identified Rad51 and ZWINT after analyzing these three genes in terms of survival (Figure 1, Figure 2b). As Rad51 has not been reported in HCC by bioinformatics, we selected it for further study.

### Rad51 expression level in HCC

**Figure 3.**
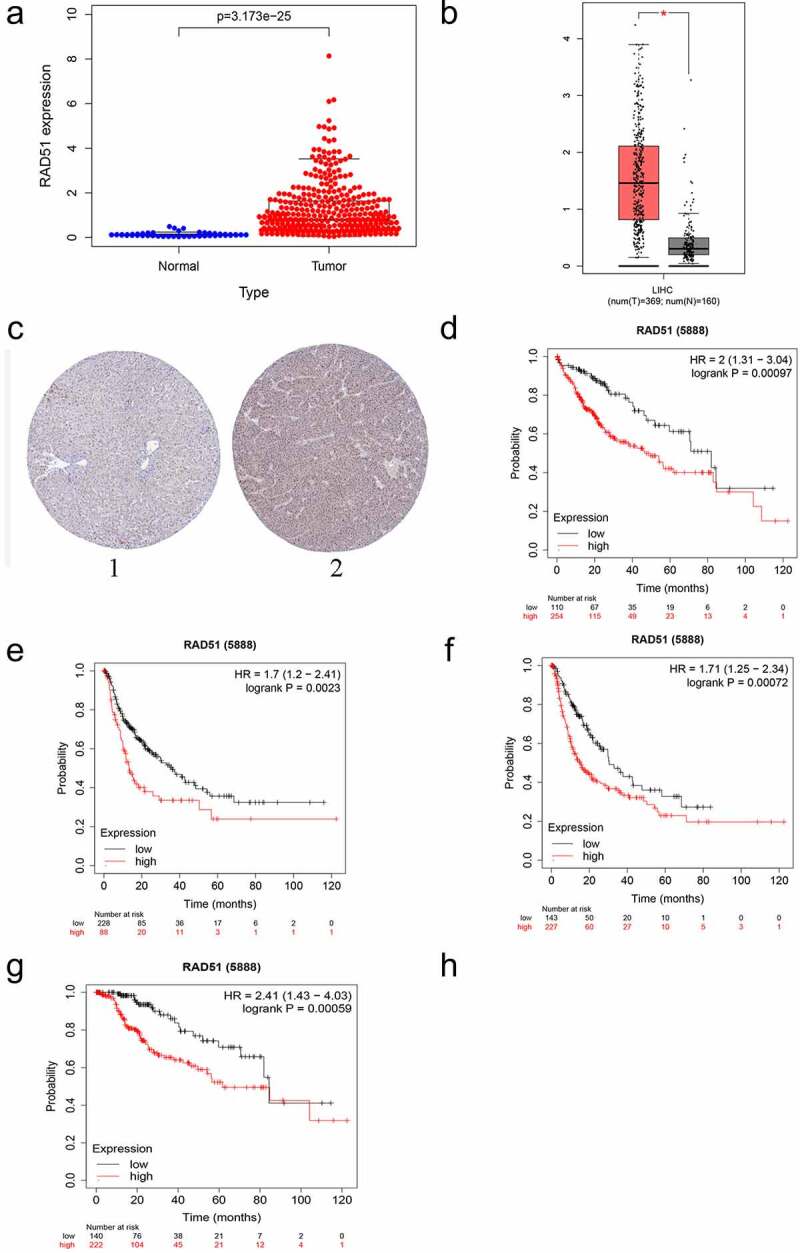
*The expression level and survival analysis of Rad51 in HCC*. A. The expression level of Rad51 in HCC from TCGA cohort. B. The expression level of Rad51 in HCC from GEPIA database. C. The protein expression of Rad51 in HCC from HPA database. D-G. Survival analysis of OS, RFS, PFS and DSS (n = 364, n = 316, n = 370, n = 362) in liver cancer

For the verification of whether Rad51 expression has an effect on patients with liver cancer, we analyzed the expression data from the TCGA database. The boxplot in Figure 3a reveals that Rad51 expression level were higher in tumor tissues in contrast to normal tissues (p-value < 3.173 × 10^−25^). Moreover, GEPIA was also used to examine Rad51 expression in HCC and healthy individuals (Figure 3b). Based on the finding, Rad51 demonstrated higher expression in HCC samples than in normal individuals. In order to better comprehend how Rad51 proteins are expressed in HCC, the HPA database was utilized to analyze Rad51 expression in HCC, and the outcomes explained that tumor tissues expressed higher protein levels of Rad51 in comparison to normal samples in HPA039310 with antibodies (Figure 3c). Besides, we employed a Kaplan-Meier plotter to examine the survival data with respect to Rad51expression in HCC (Figure 3d-g). As presented in the pictures, Rad51 expression was negatively associated with OS, PFS, RFS, and DSS in patients with liver cancer (OS: HR = 2, 95%CI = 1.31–3.04, p = 0.00097; PFS: HR = 1.71, 95%CI = 1.25–2.34, *p* = 0.00072; RFS: HR = 1.7, 95%CI = 1.2–2.41, *p* = 0.0023; DSS: HR = 2.41, 95%CI = 1.43–4.03, *p* = 0.00059).

### The impact of Rad51 on the prognostic of HCC

**Figure 4.**
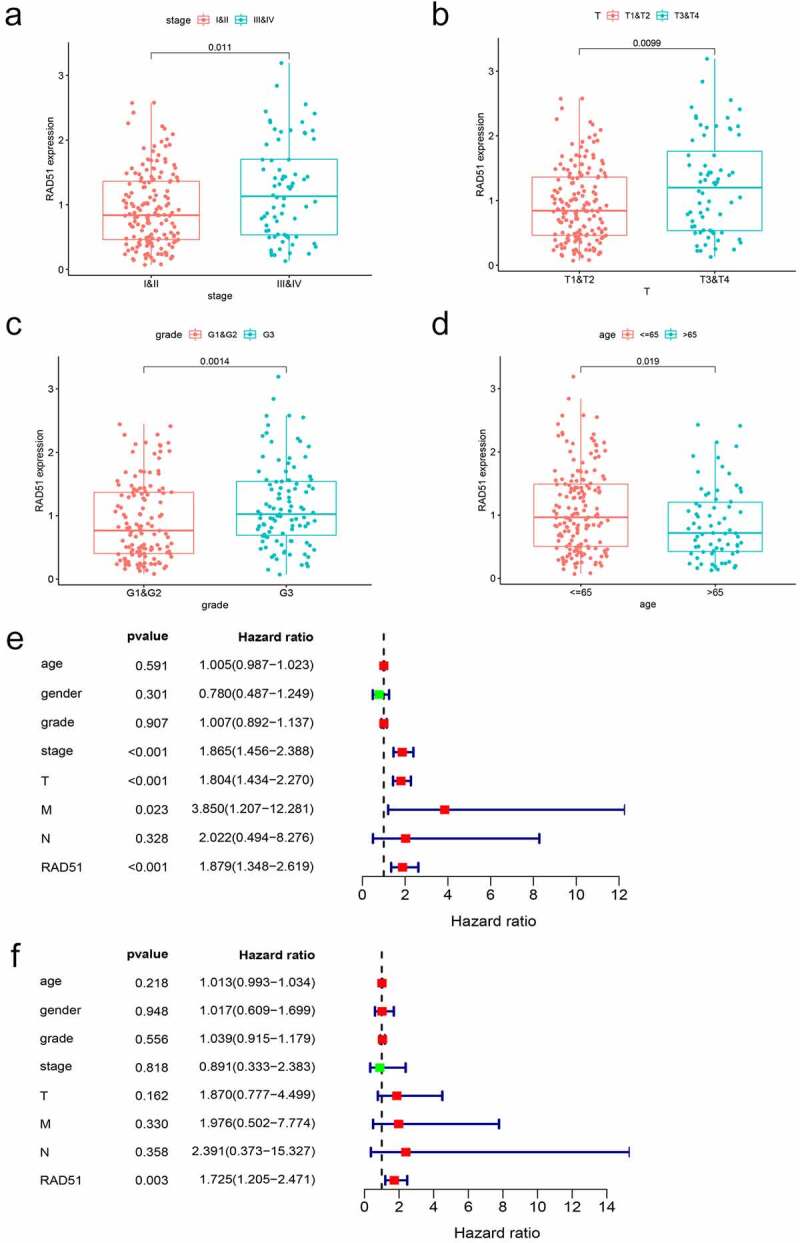
*The prognostic value of Rad51 in HCC*. Expression of Rad51 correlated significantly with clinical stage (a), pathologic T stage (b), grade (c) and age (d). Univariate COX analysis (e) and multivariate COX analysis (f) revealed the correlation between Rad51 and clinicopathological features in HCC patients

To better understand the impact of Rad51 on HCC patient outcomes, we evaluated the expression of Rad51 with respect to several clinicopathological parameters of HCC. Patients with higher Rad51 expression had more advanced tumors in the clinical stage, based on our findings (Figure 4a). As before, Rad51 expression was positively correlated with pathological T stage (Figure 4b), grade (Figure 4c), and age (Figure 4d). Subsequently, we performed univariate Cox analysis and multivariate Cox analysis to validate the reliability of the prognostic value in the HCC cohort. Univariate Cox analysis indicated that clinical stage (p-value<0.01, HR = 1.865, 95% CI = 1.456–2.388), pathological T stage (p-value<0.01, HR = 1.804, 95% CI = 1.434–2.270), pathological M stage (p-value<0.023, HR = 3.850, 95% CI = 1.207–12.281) and Rad51 expression (p-value<0.01, HR = 1.879, 95% CI = 1.348–2.619) were effective predictor for the outcomes of HCC patients (Figure 4e). Moreover, Rad51 expression was also found to be an independent prognostic factor for the HCC cohort in multivariate cox analysis (figure 4f). Besides, Kaplan-Meier analysis was plotted to examine the correlation of Rad51 expression with clinicopathological variables, such as stage, grade, AJCC_T, vascular invasion, gender, race, alcohol consumption, and hepatitis virus. Increased Rad51 expression was associated with poorer OS and RFS, based on the findings, in stage, grade, patients without vascular invasion, patients without hepatitis virus, white, Asian, male, and female (Table 1). To put it another way, these clinicopathological variables are closely related to the outcome of patients with high Rad51 expression.

**Table 1.** Kaplan-Meier plotter to determine the effect of different clinicopathologic variables on the expression of Rad51 gene and clinical prognosis in HCC

Clinicopathological characteristics	Overall survival (OS)			Relapse free survival (RFS)		
	N	HR	P	N	HR	P
Stage						
1	170	1.87(0.93–3.73)	0.0735	153	1.76(1.02–3.02)	0.04
2	83	4.84(1.45–16.16)	*	74	0.65(0.33–1.31)	0.23
3	83	2.13(1.09–4.16)	0.024	68	2.14(1.07–4.27)	0.028
4	54	– –	– –	0	– –	– –
Grade						
1	55	3.04(1.16–7.97)	0.018	45	2.76(0.99–7.69)	0.044
2	174	2.01(1.07–3.8)	0.028	147	2.23(1.32–3.76)	*
3	118	1.72(0.88–3.34)	0.11	106	1.74(0.97–3.14)	0.059
4	12	– –	– –	11	– –	– –
ATCC						
1	180	1.81(0.95–3.45)	0.7	160	1.85(1.07–3.2)	0.026
2	90	3.73(1.32–10.85)	*	79	1.57(0.76–3.17)	0.2
3	78	1.70(0.84–3.41)	0.13	65	1.81(0.91–3.59)	0.085
4	13	– –	– –	6	– –	– –
Vascular invasion						
N	203	1.93(1.08–3.44)	0.023	175	1.49(0.89–2.5)	0.13
micro	90	1.57(0.7–3.51)	0.27	81	2.22(1.12–4.4)	0.019
macro	16	– –	– –	14	– –	– –
Gender						
male	246	2.83(1.59–5.05)	**	208	1.73(1.15–2.62)	*
female	118	1.84(1.05–3.23)	0.032	105	1.78(0.99–3.21)	0.05
Race						
white	181	1.58(0.95–2.62)	0.073	147	1.96(1.24–3.11)	*
Asian	155	3.92(2.05–6.77)	***	143	2.66(1.57–4.51)	**
black	17	– –	– –	13	– –	– –
Alcohol consumption						
yes	115	2.02(1.02–4.02)	0.04	98	3.21(1.48–6.93)	*
no	202	2.02(1.25–3.25)	*	182	1.72(1.09–2.72)	0.019
HBV						
yes	150	1.48(0.72–3.06)	0.29	138	1.39(0.8–2.4)	0.24
no	167	2.37(1.5–3.74)	**	142	2.45(1.47–4.08)	**

### The relationship of Rad51 expression level with immune infiltration in patients with liver cancer.

**Figure 5.**
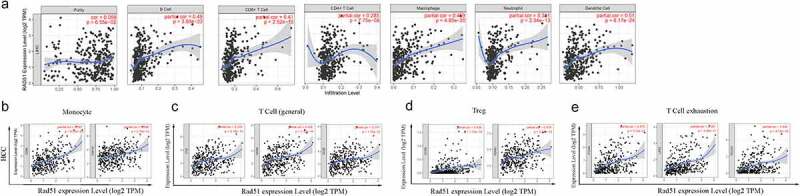
*The relationship between Rad51 expression levels and immune filtrates in HCC*. A. Rad51 expression level is significantly positive correlated with immune cells. Correlation analysis between Rad51 expression and immunological markers in HCC: B. Monocyte; C. T cell (general); D. Treg; E. T cell exhaustion

Immune infiltrations have a significant role in the formation and progression of HCC [20]. The link between Rad51 expression and the six immune infiltrates was investigated. As can be seen from the images that Rad51 expression had considerably positively correlation with infiltration level from B cells (partial.cor = 0.49, p = 3.88 × 10^−22^), CD8 + T cells (partial.cor = 0.41, p = 2.52 × 10^−15^), CD4 + T cells (partial.cor = 0.285, p = 7.75 × 10^−8^), macrophage (partial.cor = 0.469, p = 4.80 × 10^−20^), neutrophil (partial.cor = 0.381, p = 2.34 × 10^−13^), and dendritic cell (partial.cor = 0.51, p = 6.17 × 10^−24^) in HCC, it worth noting that, Rad51 expression was also positively correlated with the tumor purity (COR = 0.099, P = 6.55 × 10^−2^, Figure 5a). The expression level of Rad51 is strongly associated with immune infiltration in HCC, as per these findings.

We adopted the TIMER database to examine the association between Rad51and immune cell markers in order to gain a better understanding of the connection between Rad51 expression and immune infiltration. After adjustment based on purity, we observed that the majority of the immune cell markers had a positive association with the Rad51 expression (Table 2). The expression of CD86 and CD115 on monocytes, CD3D, CD3E, and CD2 on T cells, CCR8 and TGFβ on Treg, and PD-1, CTLA4, and TIM3 on exhausted T cells were all found to be strongly linked with Rad51 expression (Figure 5b-e). Furthermore, we employed the GEPIA database to verify the relationship between the expression of Rad51 and that immune cell marker in HCC. The results from the GEPIA database also revealed that the data was consistent (Table 3). Then, in the tumor microenvironment, we utilized the CIBERSORT database to investigate the disparities among the Rad51 high expression group and low expression group. T cells CD4 memory, T cells follicular helper, T cells regulatory (Tregs), Monocytes, Macrophages M0, and Mast cells resting exhibited significant differences between the two groups (Supplementary Figure 1). Finally, we looked into the relationship between Cancer-associated fibroblast (CAF) and Rad51 expression. By using the EPIC, MCPCOUNTER, and XCELL algorithms, we found that CAF infiltration level was closed related to the expression profile of RAD51 in 16 of the 40 cancers from the TCGA database, with HCC being one of them (Supplementary Figure 2). Overall, Rad51 shows a substantial correlation with immune infiltration and the mechanism must be explored in further work.

**Table 2.** Correlation analysis between Rad51 and relate genes and markers of immune cells in TIMER

Description	Gene markers	None		Purity	
		Cor	P	Cor	p
B cell	CD19	0.31	***	0.351	***
	CD79A	0.224	***	0.299	***
Monocyte	CD86	0.397	***	0.507	***
	CD115(CSF1R)	0.262	***	0.366	***
T cell(general)	CD3D	0.346	***	0.426	***
	CD3E	0.365	***	0.371	***
	CD2	0.282	***	0.379	***
TAM	CCL2	0.147	*	0.216	***
	CD68	0.268	***	0.323	***
	IL10	0.285	***	0.362	***
M1	INOS(NOS2)	−0.041	0.432	0.037	0.48
	IRF5	0.372	***	0.367	***
	COX2(PTGS2)	0.123	0.019	0.2	**
M2	CD163	0.164	*	0.234	***
	VSIG4	0.193	**	0.266	***
	MS4A4A	0.187	0.029	0.099	0.06
Neutrophils	CD66 b(CEACAM8)	0.103	0.048	0.128	0.01
	CD11b(ITGAM)	0.366	***	0.434	***
	CCR7	0.121	0.02	0.208	***
Natural killer cell	KIR2DL1	0.01	0.84	0.025	0.64
	KIR2DL3	0.165	*	0.194	**
	KIR2DL4	0.261	***	0.272	***
	KIR3DL1	0.021	0.68	0.022	0.67
	KIR3DL2	0.126	0.01	0.14	*
	KIR3DL3	0.065	0.21	0.05	0.35
	KIR2DS4	0.097	0.06	0.097	0.07
Dendritic cell	HLA-DPB1	0.266	***	0.347	***
	HLA-DQB1	0.248	***	0.316	***
	HLA-DRA	0.255	***	0.332	***
	HLA-DRA1	0.223	***	0.304	***
	BCDA-1(CD1C)	0.129	0.01	0.184	**
	BDCA-4(NRP1)	0.203	***	0.215	***
	CD11c(ITGAX)	0.372	***	0.461	***
Th1	T-bet(TBX21)	0.12	0.01	0.193	**
	STAT4	0.298	***	0.331	***
	STAT1	0.398	***	0.421	***
	IFN-γ(IFNG)	0.317	***	0.368	***
	TNF-α(TNF)	0.302	***	0.394	***
Th2	GATA3	0.26	***	0.353	***
	STAT6	0.117	0.02	0.108	0.04
	STAT5A	0.369	***	0.397	***
	IL13	0.091	0.07	0.089	0.09
Tfh	BCL6	0.089	0.07	0.096	0.07
	IL21	0.15	*	0.194	**
Th17	STAT3	0.092	0.07	0.102	0.05
	IL17A	0.052	0.31	0.063	0.24
Treg	FOXP3	0.172	**	0.229	***
	CCR8	0.365	***	0.426	***
	STAT5B	0.189	**	0.186	**
	TGFβ(TGFB1)	0.307	***	0.378	***
T cell exhaustion	PD-1(PDCD1)	0.369	***	0.444	***
	CTLA4	0.405	***	0.479	***
	LAG3	0.364	***	0.381	***
	TIM-3(HAVCR2)	0.403	***	0.517	***
	GZMB	0.168	*	0.2	**

**Table 3.** Correlation analysis between Rad51 and relate genes and markers of monocyte, T(general), Treg and T Cell exhaustion in GEPIA

	tumor		normal	
	R	P	R	P
monocyte				
CD86	0.55	***	0.4	***
CD86(CSFIR)	0.39	***	0.46	***
T(general)				
CD3D	0.5	***	0.3	***
CD3E	0.36	***	0.22	*
CD2	0.41	***	0.19	0.016
Treg				
FOXP3	0.28	***	0.13	0.11
CCR8	0.48	***	0.19	0.017
STAT5B	0.086	0.06	0.15	0.064
TGFβ(TGFB1)	0.38	***	0.4	***
T Cell exhaustion				
PD-1(PDCD1)	0.4	***	0.28	**
CTLA4	0.47	***	0.26	*
TIM3(HAVCR2)	0.42	***	0.41	***

### Functional enrichment analysis of DCGs of Rad51 in HCC

**Figure 6.**
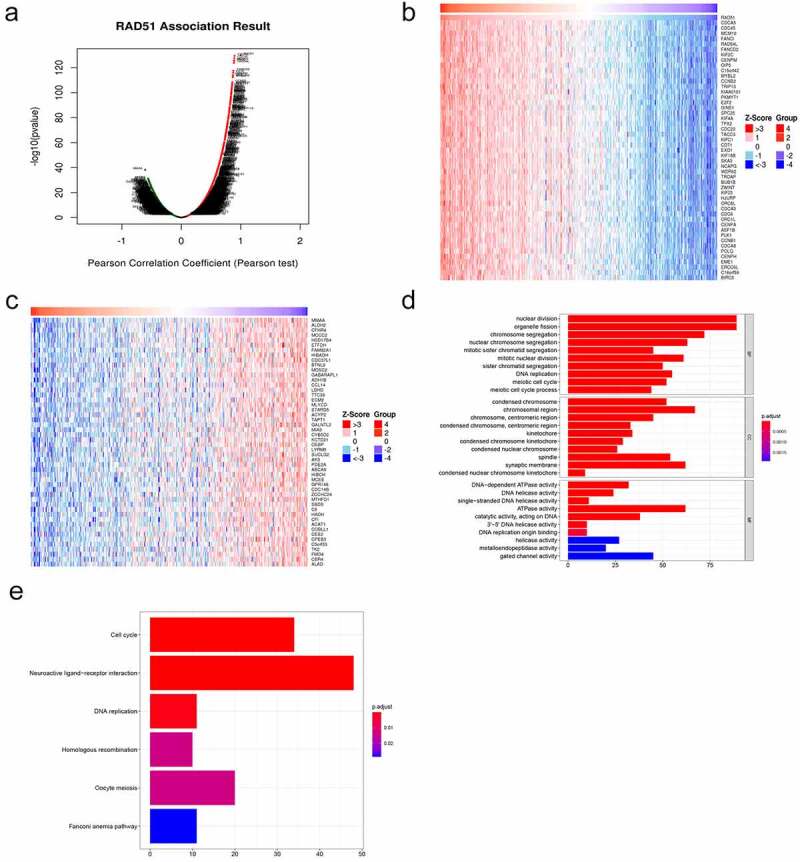
*Functional enrichment analysis of differentially co-expressed genes of Rad51 in HCC*. A. Volcano plots of differentially co-expressed genes of Rad51. The heatmap visualized the top 50 positively (b) and negatively (c) differentially co-expressed genes. GO terms of biological process (d), cell component (e) and molecular function(f)

In order to explore the potential mechanism by which Rad51 functions in HCC, we identified 11,781 DCGs from the LinkedOmics database, including 8011 positively correlated and 3770 negatively correlated genes (Figure 6a). A heatmap was used to display the top 50 DCGs that are positively related to Rad51 (Figure 6b-c). These correlated genes were chosen to conduct GO and KEGG enrichment analyses to investigate the significant biological functions and pathways. As can be seen from images that at biological process level, the GO analysis determined that the DCGs were primarily involved in the nuclear division, organelle fission, chromosome segregation, and nuclear chromosome segregation. The chromosomal region, synaptic membrane, spindle, and condensed chromosome are the most enriched categories, according to the cellular component enrichment analysis. At the molecular function level, ATPase activity, gated channel activity, catalytic activity, acting on DNA, and DNA-dependent ATPase activity were the most enriched categories (Figure 6d). The KEGG enrichment analysis illustrated that the various biological pathways are regulated by DCGs, such as neuroactive ligand-receptor interaction, cell cycle, DNA replication, homologous recombination, oocyte meiosis, and Fanconi anemia pathway (Figure 6e). Surprisingly, it was confirmed by the studies that Rad51 participates in the homologous recombination of DNA by interacting with RPA and Rad52 [21].

## Discussion

HCC, a highly malignant disease, is the fourth most prevalent malignancy and the second leading cause of cancer mortality in China [22]. Surgery is still the first line of treatment for people with liver cancer [23]. Nevertheless, the recurrence rate of patients after surgical resection is as high as 60% to 70% in 5 years [24]. Chemotherapy is the thus the preferred treatment option for patient who are likely to have poor outcomes following surgical resection as well as those who are unable to undergo surgical resection. Radiotherapy, chemotherapy, and metabolic byproducts can lead to DNA damage, which in turn leading to genomic instability and malignant transformation, and DNA repair genes can successfully counteract this threat [25]. DNA repair genes are associated with various cancers, such as Zhao, et al verified that ALKBH, a DNA repair gene, has a substantial role in preventing alkylating DNA damage and enhancing the genomic stability in pancreatic cancer cells [26]. Experiments have shown that high expression levels of BRCA promote biological behaviors such as proliferation and metastasis in breast cancer [27]. RAD51, a gene, which is located on chromosome 15q15.1, interacts with BRCA1 and BRCA2 and helps the cellular response to DNA damage [28]. When DNA is damaged, Rad51 uses homologous recombination to complete the repair of double bong breakage by integrating with single-strand DNA. Furthermore, Rad51 dysregulation has also been linked to variety of tumors, including pancreatic cancer [29], colorectal cancer [30], and ovarian cancer [31]. The DNA repair system is hyperactive in people with liver cancer, which improves the ability of cancer cells to repair DNA damage. Resultantly, the effect of chemotherapy is not satisfactory. Thus, value of Rad51 in predicting the prognosis was investigated and the efficacy of immunotherapy in HCC was assessed.

In this study, our work indicated that Rad51 has a higher expression level in liver cancer tissues in contrast to normal tissues. The protein expression of Rad51 corresponded with the aforementioned findings as well. In addition, patients with HCC who have higher Rad51 expression had poorer OS, PFS, RFS, and DSS. Univariate Cox analysis indicated that clinical stage, T stage, M stage, and Rad51 expression all have considerable value to predict the outcomes of HCC patients. Based on the multivariate Cox analysis, Rad51 was demonstrated to be an independent prognostic factor for the HCC cohort.

The immunological microenvironment of the liver, which is rich in innate and innate-like immune cells, is exceedingly complex. The chronic inflammatory response is believed to be a major driver of the development of HCC [32–34]. Chen et al. revealed that the tumor infiltration of CD8 + T lymphocytes, B cells, and dendritic cells predicts a good prognosis, while the cancer-associated fibroblast was predictive for poor prognosis [35]. It was indicated by our work that Rad51 has a considerable relationship with the immune cells, particularly in B cells, CD8 + T cells, macrophage, dendritic cells and cancer-associated fibroblast. Immune cells facilitate tumor growth through immune escape by upregulating immune checkpoints and proinflammatory cytokines. It has been revealed that the host immune system can build efficient antitumor immunity against tumor antigens when the immunological checkpoint is blocked [36]. Therefore, immunotherapy has emerged as a viable treatment option for individuals with advanced HCC. It has been observed that Rad51 levels in HCC have a significantly positive correlation with the levels of markers of T cell exhaustion, such as PD-1, CTLA4, and TIM3. These markers were crucial inhibitory immune checkpoint proteins, allowing tumor cells to evade immune surveillance. This suggests that Rad51 plays a key role in inducing the exhaustion of T cells, and that the upregulation of the markers strengthens the suppression of antitumor immunity. It has been further discovered that the majority of the markers are correlated with Rad51. This implies that Rad51 might play an important role in regulating and recruiting the infiltrating immune cells in HCC. In order to explore the function of Rad51 in HCC GO and KEGG analyses were performed. The DCGs of Rad51, according to GO analysis, were largely implicated in the nuclear division. In addition, the cell-cycle pathway was identified as the critical pathway through KEGG analysis.

This study is the first to validate the role of Rad51 in the immune microenvironment of liver cancer employing a bioinformatics approach. Nevertheless, our study still has limitations, we identified Rad51 from the ICGC database and verified the expression level of Rad51 using the TCGA database, but further clinical trials required for verifying our findings.

## Conclusion

To sum up, the high expression of Rad51 is correlated with poor outcomes in HCC. Further studies on the correlation among Rad51 expression and immune infiltration revealed that Rad51 may be a novel target of immunotherapy in HCC.

## Supplementary Material

Supplemental Material

## Highlights

1. The expression of Rad51 was related with prognosis of HCC patients.
2. Rad51 has a significant correlation with immune infiltrations.
3. Rad51 may be a novel target of immunotherapy in HCC.
