# Supplementary figures and images for: Identification of Rad51 as a prognostic biomarker correlated with immune infiltration in hepatocellular carcinoma
Source: Bioengineered. 2021 Jun 11;12(1):2664–75. doi: 10.1080/21655979.2021.1938470 (PMC8806544; doi:10.1080/21655979.2021.1938470)

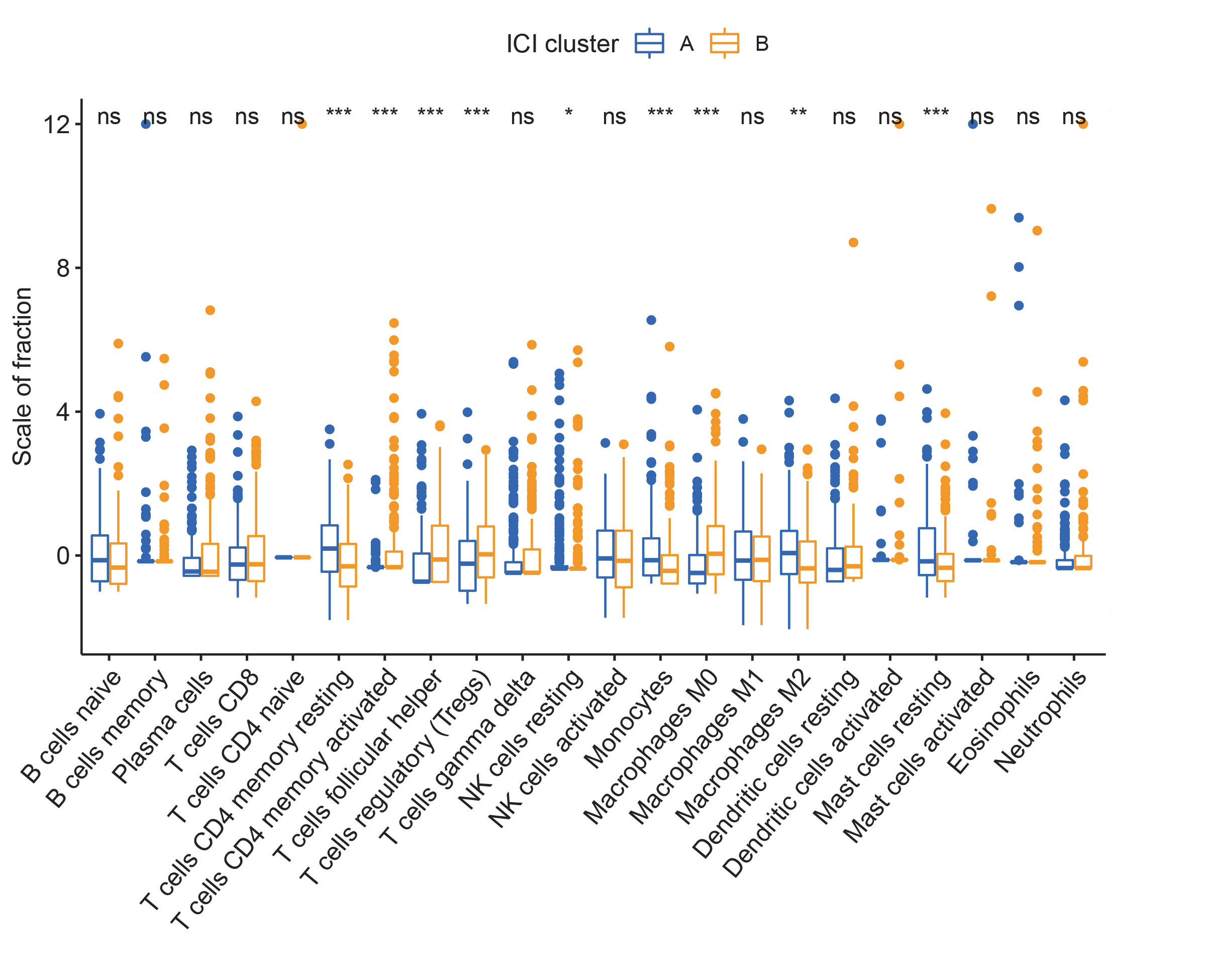

Supplement: Supplemental Material [file KBIE_A_1938470_SM1843.zip › supplementary/Supplement_Fig_1.png]

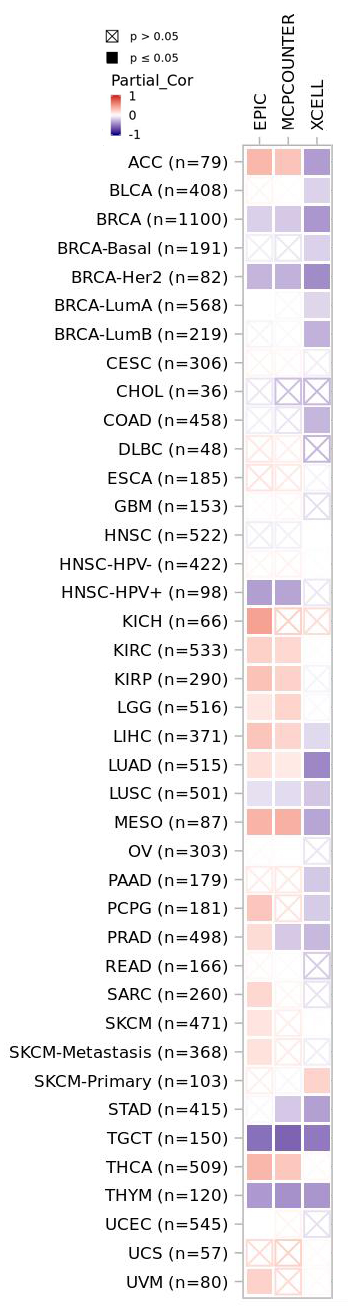

Supplement: Supplemental Material [file KBIE_A_1938470_SM1843.zip › supplementary/Supplement_Fig_2.png]
